# Peanut-specific T cell responses in patients with different clinical reactivity

**DOI:** 10.1371/journal.pone.0204620

**Published:** 2018-10-10

**Authors:** Giovanni Birrueta, Victoria Tripple, John Pham, Monali Manohar, Eddie A. James, William W. Kwok, Kari C. Nadeau, Alessandro Sette, Bjoern Peters, Véronique Schulten

**Affiliations:** 1 La Jolla Institute for Allergy & Immunology, La Jolla, CA, United States of America; 2 Department of Medicine, Division of Pulmonary and Critical Care Medicine, Sean N. Parker Center for Allergy and Asthma Research, Stanford University School of Medicine, Stanford, CA, United States of America; 3 Benaroya Research Institute at Virginia Mason, Seattle, WA, United States of America; 4 Department of Medicine, University of Washington, Seattle, WA, United States of America; 5 Department of Medicine, University of California San Diego, La Jolla, CA, United States of America; Monash University, AUSTRALIA

## Abstract

Whole extract or allergen-specific IgE testing has become increasingly popular in the diagnosis of peanut allergy. However, much less is known about T cell responses in peanut allergy and how it relates to different clinical phenotypes. CD4+ T cells play a major role in the pathophysiology of peanut allergy as well as tolerance induction during oral desensitization regimens. We set out to characterize and phenotype the T cell responses and their targets in peanut sensitized patients. Using PBMC from peanut-allergic and non-allergic patients, we mapped T cell epitopes for three major peanut allergens, Ara h 1, 2 and 3 (27 from Ara h 1, 4 from Ara h 2 and 43 from Ara h 3) associated with release of IFNγ (representative Th1 cytokine) and IL5 (representative Th2 cytokine). A pool containing 19 immunodominant peptides, selected to account for 60% of the total Ara h 1-3-specific T cell response in allergics, but only 20% in non-allergics, was shown to discriminate T cell responses in peanut-sensitized, symptomatic vs non-symptomatic individuals more effectively than peanut extract. This pool elicited positive T cell responses above a defined threshold in 12/15 sensitized, symptomatic patients, whereas in the sensitized but non-symptomatic cohort only, 4/14 reacted. The reactivity against this peptide pool in symptomatic patients was dominated by IL-10, IL-17 and to a lesser extend IL-5. For four distinct epitopes, HLA class II restrictions were determined, enabling production of tetrameric reagents. Tetramer staining in four donors (2 symptomatic, 2 non-symptomatic) revealed a trend for increased numbers of peanut epitope-specific T cells in symptomatic patients compared to non-symptomatic patients, which was associated with elevated CRTh2 expression whereas cells from non-symptomatic patients exhibited higher levels of Integrin β7 expression. Our results demonstrate differences in T cell response magnitude, epitope specificity and phenotype between symptomatic and non-symptomatic peanut-sensitized patients. In addition to IgE reactivity, analysis of peanut-specific T cells may be useful to improve our understanding of different clinical manifestations in peanut allergy.

## Introduction

Peanut allergy (PA) is among the most common food allergies and its prevalence has increased over time [1]. In developed countries, PA has been reported to affect up to 1% of children and 0.6% of adults [2]. In contrast to milk and egg allergy, PA is not commonly outgrown [3] and is associated with severe, potentially fatal anaphylactic reactions [4]. Due to this high risk of adverse reactivity, management of the disease usually consists of strict peanut avoidance. However, this is logistically difficult to achieve and patients are at a constant risk of accidental exposure to the allergen. To minimize the risk of serious allergic reactions following accidental peanut ingestion, patients are often advised to carry self-injectable epinephrine. The burden of constant food avoidance and fear of accidental ingestion can have a significant impact on the quality of life of the patients [5].

Extensive studies over the last decades have significantly improved our knowledge of IgE reactivity against peanut and its individual components [6–9]. Indeed, common clinical diagnostic tests are based on measuring peanut-specific IgE titers or skin test reactivity, which provide evidence of allergic sensitization and are usually indicative of clinical reactivity. Compared to antibodies, much less is known about the peanut-specific allergic T cell response and its association with clinical symptoms. T cell epitopes have been identified for the major allergens Ara h 1 [10–12] (7S vicillin-like globulin) and Ara h 2 [13–15](2S albumin) but the molecular targets for other peanut allergens remain unknown.

The presence of peanut-specific IgE antibodies is not always associated with clinical peanut allergy. In 2010, Flinterman et al. examined peanut-specific T cell responses in peanut sensitized, allergic and non-allergic individuals, reporting readily detectable responses in both cohorts[16]. Little is known about potential differences in the T cell epitope repertoire or the phenotype of peanut-specific T cells in symptomatic versus non-symptomatic patients.

With the present study, we sought to identify T cell epitopes derived from peanut allergens Ara h 1, 2 and 3, as it has been reported that clinical symptoms were mostly associated with IgE reactivity to Ara h 1, 2 and 3 [9] in American patients. Moreover, cytokine polarization (IL-5 vs IFNγ production) of peanut-specific T cell responses was analyzed in peanut-sensitized allergic and non-sensitized individuals. In a second line of analysis, our objective was to determine if we could detect quantitative and/or qualitative differences in peanut-specific T cell responses between peanut-sensitized and symptomatic versus peanut-sensitized non-symptomatic patients. Lastly, HLA restrictions of antigenic peptides were used to facilitate the design of tetramer reagents to characterize the phenotype of peanut-specific T cells in symptomatic versus non-symptomatic patients. Tetramer reagents represent a valuable tool for monitoring the surface phenotype, function and frequency of allergen-specific T cells at the single cell level [17]. Accordingly we sought to leverage our epitope identification studies to enable development of tetramer staining reagents. These reagents may contribute significant insights into the differences of a non-symptomatic, tolerant immune response versus the adverse, potentially fatal allergic reaction associated with clinical peanut allergy.

## Materials and methods

### Study population and PBMC isolation

A cohort of 50 patients was recruited from Stanford and San Diego, CA, following Institutional Review Board approval by the La Jolla Institute’s Institutional Review Board and Stanford University’s Institutional Review Board (IRB protocols: IRB-8629, VD-112-0217). Patients 18 or older enrolled in this study provided written consent. Patients below the age 18 provided oral assent and their parent or guardian provided written consent. Demographic and clinical information is summarized in **Table 1**. The non-allergic cohort is 70% female with a median age of 37, the symptomatic cohort is 58% female with a median age of 23 and the non-symptomatic cohort is 29% female with a median age of 33. The differences in age between the symptomatic versus non-allergic cohort and symptomatic versus non-symptomatic cohorts are statistically significant (p = 0.003, p = 0.02, respectively) as determined by two-tailed Mann-Whitney test. Peanut-specific IgE titers were determined from plasma using the ImmunoCAP (Thermo Fisher, Uppsala, Sweden). Clinical allergy was determined either by oral food challenge or using a questionnaire to determine a clinical history consistent with peanut allergy. Peanut-sensitized but non-symptomatic donors were categorized based on positive IgE titers (>0.3 kU/L) and lack of clinical reactivity to peanut after peanut ingestion (**Table 1**). PBMCs were isolated from whole blood by density gradient centrifugation according to manufacturers’ instructions (Ficoll-Hypaque, Amersham Biosciences, Uppsala, Sweden) and cryo-preserved for subsequent further analysis.

**Table 1:** A summary of demographic and clinical information for all patient cohorts.

LJI	Peanut tolerated in challenge (mg)	IgE(KU/L)	Status	Peanut ingestion*
1267	n.d.**	<0.1	non-allergic	n.d.
1266	n.d.	<0.1	non-allergic	n.d.
1265	n.d.	<0.1	non-allergic	n.d.
1264	n.d.	<0.1	non-allergic	n.d.
1263	n.d.	<0.1	non-allergic	n.d.
D00013	n.d.	<0.1	non-allergic	n.d.
U00127	n.d.	<0.1	non-allergic	n.d.
U00081	n.d.	<0.1	non-allergic	n.d.
D00024	n.d.	n.d.	non-allergic	n.d.
U00059	n.d.	<0.1	non-allergic	n.d.
1274	83.6	100	symptomatic	avoidance
1273	33.6	77.2	symptomatic	avoidance
1272	7.7	480	symptomatic	avoidance
1271	83.6	>100	symptomatic	avoidance
1270	83.6	80	symptomatic	avoidance
1268	33.6	1.8	symptomatic	avoidance
1262	7.7	73.2	symptomatic	avoidance
1261	32.7	n.d.	symptomatic	avoidance
1239	32.7	18.6	symptomatic	avoidance
1238	44	100	symptomatic	avoidance
1237	1	25.9	symptomatic	avoidance
2376	n.d.	1.86	symptomatic	avoidance
2380	n.d.	1.34	symptomatic	avoidance
2381	n.d.	21	symptomatic	avoidance
2384	n.d.	100	symptomatic	avoidance
2371	n.d.	3.44	symptomatic	avoidance
2375	n.d.	1.9	symptomatic	avoidance
2377	n.d.	0.84	symptomatic	avoidance
2386	n.d.	4.35	symptomatic	avoidance
2387	n.d.	0.4	symptomatic	avoidance
2391	n.d.	2.87	symptomatic	avoidance
2420	n.d.	0.54	symptomatic	avoidance
2383	n.d.	1.75	symptomatic	avoidance
2400	n.d.	4.45	symptomatic	avoidance
2422	n.d.	0.84	symptomatic	avoidance
2379	n.d.	0.31	symptomatic	avoidance
1381	n.d.	1.84	non-symptomatic	sometimes
1864	n.d.	51.20	non-symptomatic	sometimes
1198	n.d.	6.53	non-symptomatic	n.d.
1437	n.d.	0.81	non-symptomatic	n.d.
2017	n.d.	1.36	non-symptomatic	rarely
1440	n.d.	1.16	non-symptomatic	n.d.
1453	n.d.	0.52	non-symptomatic	n.d.
2021	n.d.	1.39	non-symptomatic	sometimes
2036	n.d.	1.86	non-symptomatic	often
2145	n.d.	0.46	non-symptomatic	rarely
2201	n.d.	0.42	non-symptomatic	sometimes
2235	n.d.	0.47	non-symptomatic	sometimes
1514	n.d.	1.14	non-symptomatic	n.d.
2020	n.d.	0.75	non-symptomatic	sometimes

*rarely—once a month or less

*sometimes- weekly or multiple times per month

*often daily or multiple times a week

** n.d.- not determined

### Peanut extract, peptide selection and sythesis

Peanut extract was obtained from Greer (Lenoir, NC). A total of 5 peanut (*Arachis hypogaea*) protein sequences, corresponding to Ara h 1.0101, Ara h 2.0101, Ara h 2.0201, Ara h 3.0101 and Ara h 3.0201 were considered. The sequences were aligned and grouped into 3 different clusters, corresponding to Ara h 1, 2 and 3. Next, 15-mer peptides, overlapping by 10 residues, were generated for all sequences. Redundant 15-mers were removed, leaving a set of 386 unique peptides. An additional 14 peptides were added to cover gap regions formed by the alignment. This resulted in a final set of 400 15-mer peptides. This set was synthetized by Synthetic Biomolecules (San Diego, CA) as crude material on a 1 mg scale (purity of >70%), and utilized for subsequent experiments. Representative peptides from this set were randomly selected and tested for quality control by HPLC and mass spectrometric analysis to confirm purity and sequence identity. Peptides were reconstituted at 40 mg/ml in DMSO. Reconstituted peptides were stored at -20°C. In performed assay, the DMSO concentration added to the culture did not exceed 0.25%.

### T cell *in vitro* culture expansion

For *in vitro* expansions, PBMCs were thawed and cultured in RPMI 1640 (Omega Scientific, Tarzana, CA) supplemented with 5% human AB serum (Gemini Bio-Products, West Sacramento, CA) at a density of 2 × 10^6^ cells per mL in 24-well plates (Corning, San Diego, CA) and stimulated with peanut extract (10 μg/ml) or peptide pools at 5 μg/mL. Cells were kept at 37°C, 5% CO_2_, and additional IL-2 (10 U/mL; ThermoFisher, San Diego, CA) was added every 3 days after initial antigenic stimulation. On day 14, cells were harvested and reactivity against peanut extract (10 μg/ml), peanut specific peptide pools (5 μg/ml) and individual peptides (10 μg/ml) was assessed.

### Dual ELISPOT assays

The production of IL-5 and IFNγ post-stimulation with peanut extract was analyzed in dual ELISPOT assays. Flat-bottom 96-well nitrocellulose plates (Millipore, Bedford, MA) were prepared according to the manufacturer’s instructions and coated with either 10 μg/ml of both anti-human IL-5 (clone TRFK5) and anti-human IFNγ (clone 1-D1K) or anti-human IL-10 (clone 9D7) and anti-human IL-17 (clone MT44.6) (Mabtech, Cincinnati, OH). PBMC were then incubated at a density of 1 × 10^5^/well either with peptide pools (5 μg/ml) or individual peptides (10 μg/ml), peanut extract (10 μg/ml), PHA (10 μg/ml), or medium containing 0.25% DMSO (corresponding to the highest percentage of DMSO in the pools/peptides condition) as a control. After 24 h, cells were removed, and plates were incubated with a cocktail containing either biotinylated anti-human IL-5 (1:50 dilution) (clone 5A10) and horseradish peroxidase (HRP)-conjugated anti-human IFNγ (1:200 dilution) (clone 7-B6-1) or biotinylated anti-human IL-17 (1:200 dilution) (clone MT504) anti-human alkaline phosphatase (AP)-conjugated IL-10 (1:200 dilution) (clone 12G8) (Mabtech, Cincinnati, OH) at 37°C. After 2 h, spots corresponding to the biotinylated IL-5 Ab were developed by incubation with Alkaline-phosphatase-Complex (Vector Laboratories, Burlingame, CA) and then visualized by applying the Vector Blue Alkaline Phosphatase Substrate Kit III (Vector Laboratories, Burlingame, CA) according to the manufacturer’s instructions. Spots corresponding to the HRP-conjugated anti-human IFNγ Ab were visualized by incubation with 3-amino-9-ethylcarvazole solution (Sigma-Aldrich, St. Louis, MO). Spots were counted by computer-assisted image analysis (KS-ELISPOT reader, Zeiss, Munich).

Each assay was performed in triplicate. The level of statistical significance was determined with a Student *t* test using the mean of triplicate values of the response against relevant pools or individual peptides versus the response against the DMSO control. Criteria for response positivity were 20 spot-forming cells (SFCs)/10^6^ PBMC, *p* ≤ 0.05, and a stimulation index (SI) ≥ 2. SFCs are measured per 10^5^ PBMC, subsequently readings are background subtracted and multiplied by 10 to be expressed as SFC per million PBMC.

### Tetramer staining with column enrichment and flow cytometry

Whole PBMC (2 × 10^6^) were stimulated for 2 weeks *in vitro* with 15-mer peanut allergen-derived antigenic epitopes. After 14 days, cells were washed and co-stained with phycoerythrin (PE)-labeled pMHC II tetramer (MHC mismatch used as negative control), loaded with the respective peanut-derived epitope. Staining was performed at 37°C for 2 h. After staining, cells were washed and labeled with anti-PE microbeads (Miltenyi, Auburn, CA) following by magnetic column enrichment (Miltenyi, Auburn, CA). After column enrichment, the cells were washed and stained with a cocktail containing anti-CD4 (APCef780, clone RPA-T4, ThermoFisher, San Diego, CA), anti-CD3 (AF700, clone UCHT1, ThermoFisher, San Diego, CA), anti-CD8/CD14/CD19 (V500, clones RPA-T8, M5E2, H1B19, BD, San Diego, CA), anti-Integrin β7 (FitC, clone FIB504, ThermoFisher, San Diego, CA) and anti-CRTh2 (Ax647, clone BM16, Biolegend, San Diego, CA) was added. Cells were analyzed by flow cytometry using a BD LSR-II (BD, San Diego, CA). Data were analyzed utilizing FlowJo (Tree Star, Ashland, OR).

### Statistical analysis

Statistical analysis of the data was performed using GraphPAD Prism software (La Jolla, CA). Statistical comparisons were performed by Mann-Whitney test, unpaired, non-parametric, or Wilcoxon signed-rank test, paired, non-parametric. Comparisons for analyses testing a preconceived hypothesis were performed one-tailed. Two-tailed comparisons were performed for comparisons without hypothesis.

## Results

### Identification of peanut allergen-derived T cell epitopes in allergic and non-allergic patients

T cell epitope mapping was performed to determine epitopes from the major peanut allergens Ara h 1, 2 and 3, recognized by peanut allergic (n = 11) and non-allergic (n = 10) donors. As these studies were performed with samples obtained from pediatric patients, available blood sample volumes were limiting. To overcome this limitation, small pools of predicted peptides matched to the HLA molecules expressed in each donor were tested. For each donor, class II binding affinity predictions were performed using algorithms available through the IEDB (www.iedb.org)[18], to identify the top 6 predicted binders for each of the alleles expressed at the four HLA class II loci (DRB1, DRB3/4/5, DQB1, and DPB1). Accordingly, the screening load for each donor was reduced from 400 peptides to a maximum of 48 peptides (6 peptides x ≤8 class II molecules, less for homozygous donors). The six top predicted peptides for each allele were used to generate allele-specific pools, which were in turn used to expand peanut-specific cells *in vitro*. After 14 days of culture, ELISPOT assays were used to measure reactivity upon restimulation with the respective pool and each individual peptide they contained.

**Fig 1:**
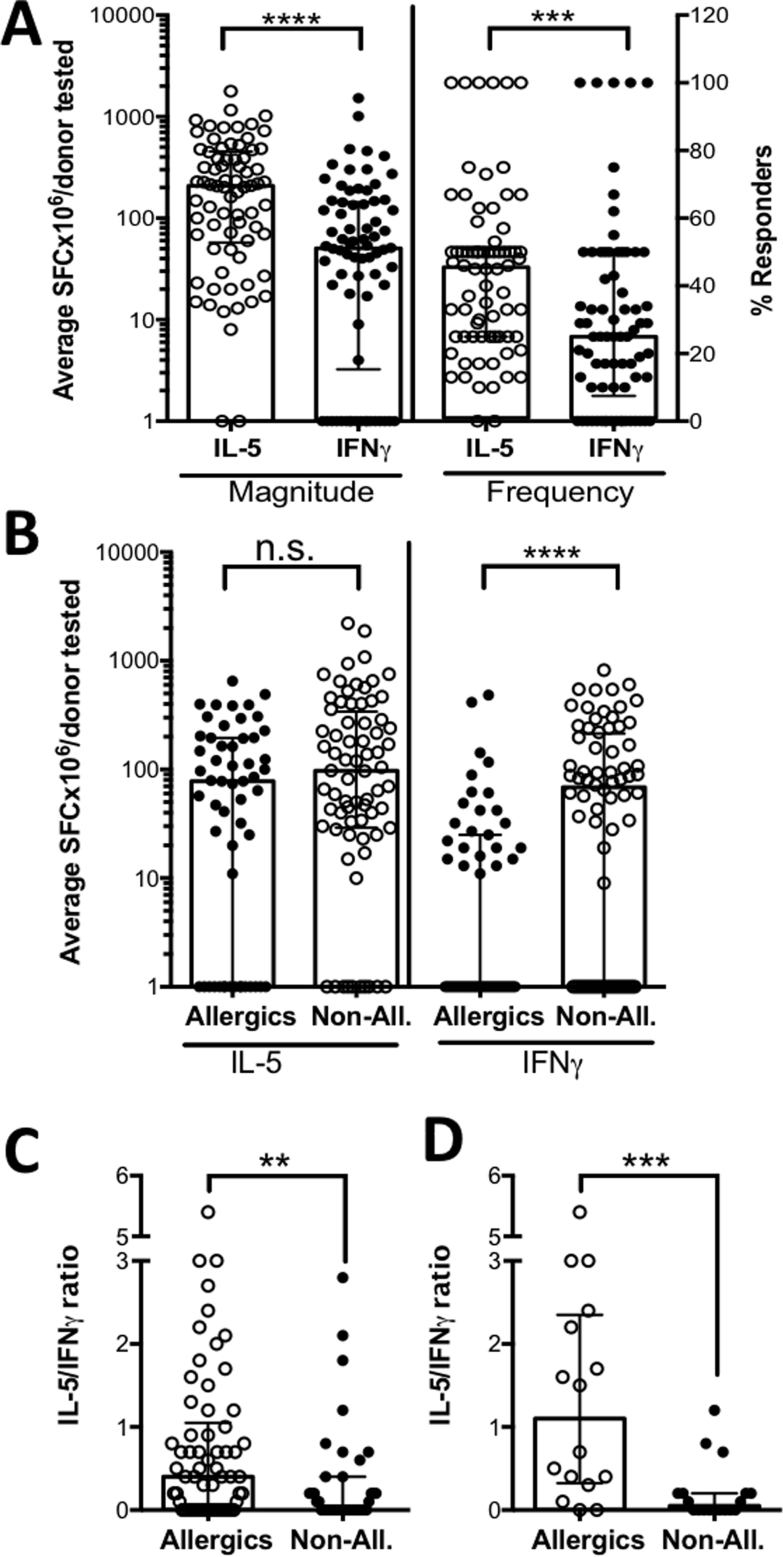
T cell reactivity against peanut allergen-derived peptides. A) Magnitude (SFC- spot forming cells per 1 PBMC million (i.e. SFC /10^6^)) and frequency (% responders) of IL-5 (open circles) and IFNγ (closed circles) production in response to individual peptides derived from Ara h 1, 2 and 3 were measured by ELISPOT in all tested donors (n = 21). Each dot represents a single peptide that elicited T cell reactivity in one or more donors. B) Magnitude of IL-5 and IFNγ production in allergics (n = 11; open circles) and non-allergic (n = 10; closed circles) patients. The response polarization, expressed as IL-5/IFNγ ratio, is shown for C) all peptides that tested positive in any given donor and D) a set of 19 selected peptides that account for 30% of the total response (60% in allergics, 20% in non-allergics, respectively). Each data point represents a single donor/peptide combination. Statistical comparison by Mann-Whitney test, two-tailed. **- p<0.01, ***-p<0.001, ****-p<0.0001.

In total, 74 out of 400 tested peptides elicited positive T cell reactivity in at least 1 donor (T cell reactivity ≥ 20 spot forming cells (SFC) for either IL-5 or IFNγ, p-value <0.05, stimulation index ≥2). A summary of the magnitude and response frequency of each individual donor/peptide combination tested is shown in **Fig 1A**. The data was also deposited in the IEDB (submission ID 1000755, URL: http://www.iedb.org/subid/1000755) Interestingly, a cohort-specific analysis of cytokine production revealed no difference in IL-5 levels in response to the peanut peptides in non-allergic compared to allergic patients. However, IFNγ production was increased significantly in non-allergic vs allergic patients (p<0.0001) (**Fig 1B**). A similar pattern is seen when directly comparing only the subset of peptides that is recognized in both cohorts (**S1 Fig**). Given this trend, we set out to investigate the overall cytokine environment of the peanut-specific T cell response in the allergic and non-allergic cohort by calculating the IL-5/IFNγ ratio. T cell responses in allergic donors had a significantly higher IL-5/IFNγ ratio compared to non-allergic donors (p = 0.0048), indicating that the response in allergic individuals is significantly more Th2-dominated compared to non-allergics (**Fig 1C**). Based on an intermediate non-formal analysis of peptide-induced IL-5 production in allergic and non-allergic donors, we selected and pooled a subset of 19 positive peptides (P19 pool) that was designed to capture a majority of the responses in allergic and less responses in non-allergic donors. Based on the complete data, this P19 pool accounts for 60% of the response in allergics and only 20% of the response in non-allergic donors. Peptides included in the P19 are highlighted in the first column of **S1 Table**. Analysis of the cytokine response polarization of the P19 pool revealed an even more significant Th2 dominance in allergics compared to non-allergics (p = 0.0006) (**Fig 1D**) than was observed for the complete set of positive peptides.

It should be noted that, due to the HLA-matched design of our epitope mapping approach, each peptide was tested in a variable number of donors and the tested peptides set was selected based on predicted binding of HLA expressed in our cohort. A full summary of the 74 positive peptides in terms of number of donors tested, response magnitude and response frequencies given in **S1 Table**.

### Differences in T cell responses between symptomatic and non-symptomatic peanut sensitized patients

Next, we were interested to assess whether T cell reactivity to the P19 pool could be used to detect differences in the T cell response between peanut-sensitized patients with and without clinically symptomatic peanut allergy. Using a new adult cohort, PBMC from patients with positive peanut-specific IgE titers (>0.3 kU_A_/L), who are either symptomatic (patients with a clinical history consistent peanut allergy, n = 15) or non-symptomatic (patients who regularly ingest peanuts without experiencing any symptoms, n = 14) were cultured *in vitro* with either peanut extract or P19 pool for 14 days. After culture, total T cell reactivity (defined here as the sum of IL-5, IL-10, IL-17 and IFNγ) in response to restimulation with peanut extract or P19 pool were determined by ELISPOT. The P19 pool was selected based on its lower reactivity in non-allergic donors, therefore it was of particular interest to investigate if it would also be less reactive in sensitized but non-symptomatic individuals or if they would exhibit responses similar to allergic, symptomatic patients.

**Fig 2:**
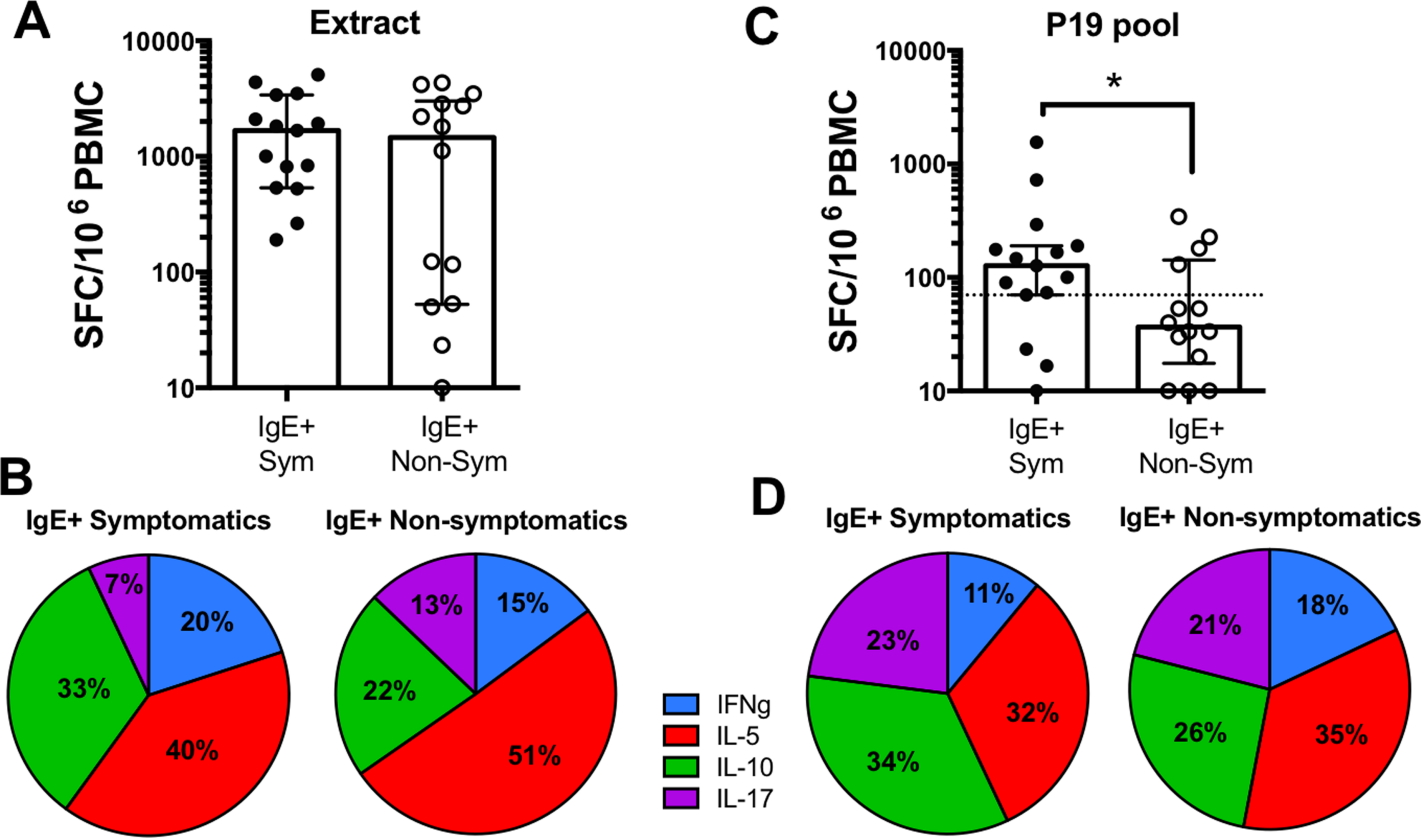
Peanut extract and epitope responses and cytokine polarization in peanut symptomatic and non-symptomatic patients. Magnitude and polarization of cytokine production (sum of IL-5, IL-10, IL-17 and IFNγ) in response to A-B) peanut extract and C-D) P19 pool is shown as SFC (spot forming cells) per 10^6^ PBMC. On panel C, dashed line indicates positive response threshold of 70 SFC. Peanut sensitization status is indicated by IgE+. Sym = symptomatic (n = 15), Non-Sym = non-symtomatic (n = 14). Statistical comparison by Mann-Whitney test, one-tailed. *- p<0.05.

A comparison of T cell responses against peanut extract did not show any differences in peanut-sensitized symptomatic patients (median 1673 SFC) compared to non-symptomatic patients (1460 SFC) (**Fig 2A**). Moreover, analysis of the cytokine polarization of peanut extract-specific T cell responses showed no significant differences between the two cohorts (**Fig 2B)**. In contrast to peanut extract, T cell reactivity against the P19 pool was significantly higher (p = 0.043) in peanut sensitized symptomatic donors (median 127 SFC) compared to sensitized, yet non-symptomatic donors (median 37 SFC) (**Fig 2C**). Again, no significant difference in cytokine polarization was detected between the two cohorts (**Fig 2D**). A more detailed analysis of the patterns of cytokine production on symptomatic and non-symptomatic donors revealed that the increased T cell response in symptomatic donors is mostly accounted for by IL-10 (p = 0.02) and IL-17 (p = 0.03). IL-5 production was modestly increased in symptomatic patients (p = 0.25) and only very little IFNγ (p = 0.5) was observed in both cohorts (**S2 Fig)**. Thus, while the use of whole peanut extract fails to reveal differences between peanut-sensitized cohorts with different clinical manifestations, differences in T cell reactivity can be detected at the epitope level. A threshold level of 70 SFC/10^6^ was artificially determined to distiguish responses in symptomatic and non-symptomatic donors. At this threshold, pool-specific T cell reactivity is detected in 80% (12/15) of the symptomatic donors. In the non-symptomatic cohort, 71% (10/14) are negative for pool-specific T cell reactivity (**Fig 2C**). Moreover, an analysis of peanut-extract specific IgE titers (listed in **Table 1**) and T cell cytokine production revealed a significant correlation for IL-5 (p<0.0001), IL-17 (p<0.0001) and IL-10 (p = 0.03) production in response to the P19 pool, whereas correlation between sIgE and extract-specific T cell responses only reach borderline significance for IL-5 (p = 0.045) and IL-17 (p = 0.043) (**Table 2**). Correlation graphs are shown in **S3 Fig**.

**Table 2:** Correlation analysis of peanut extract IgE titers and T cell cytokine production in response to peptide pool or peanut extract.

		IL-5	IFNg	IL-10	IL-17	Sum of cytokines
**Pool 19**	**R**^**2**^ **value**	0.720	0.040	0.160	0.220	0.550
	**p value**	<0.0001	0.540	0.034	0.009	<0.0001
**Extract**	**R**^**2**^ **value**	0.140	0.001	0.016	0.140	0.100
	**p value**	0.045	0.870	0.520	0.043	0.092

### Characterization of peanut-specific T cell phenotypes in symptomatic and non-symptomatic donors using tetramer reagents

**Fig 3:**
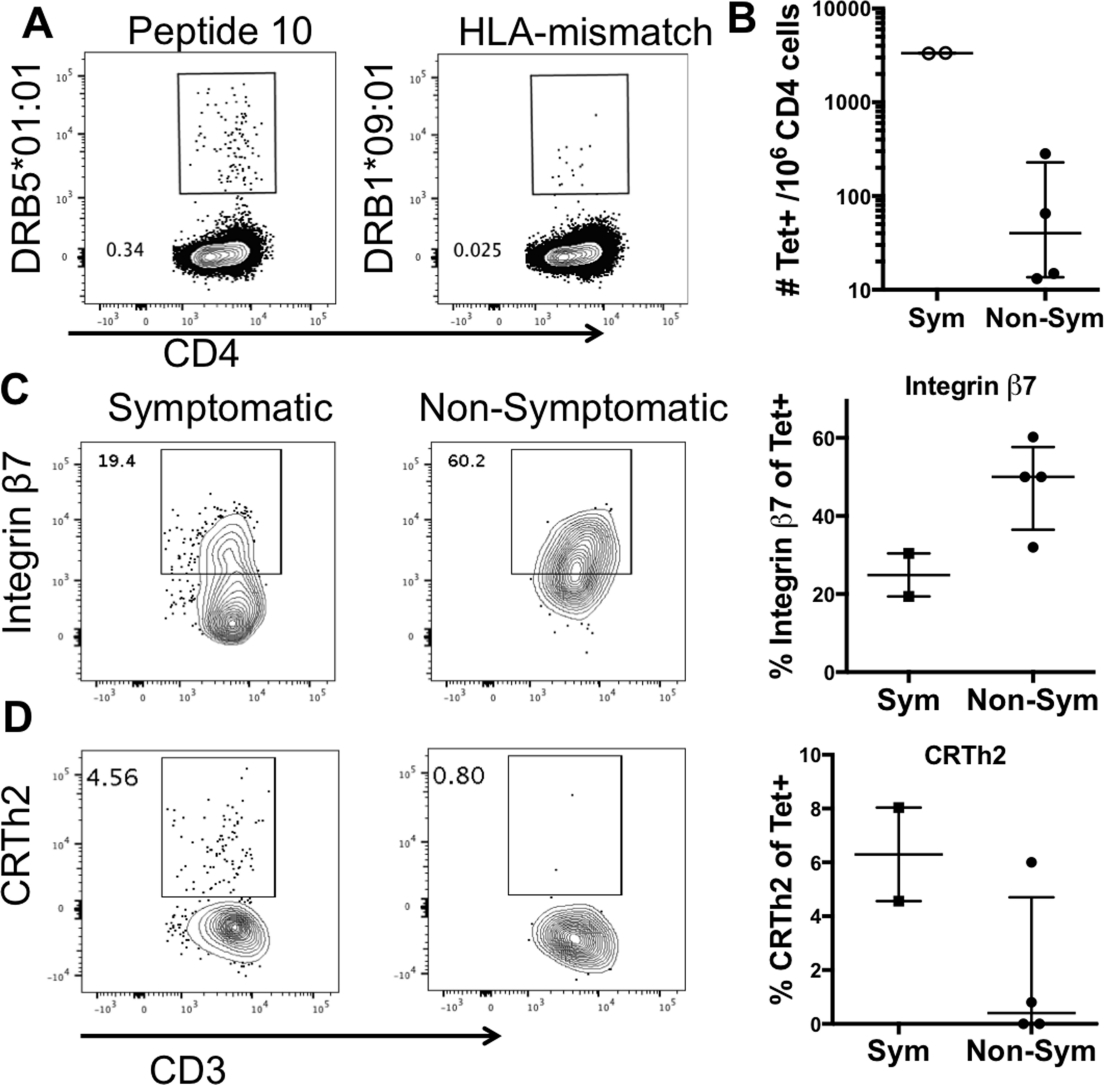
Tetramer staining and phenotypic surface marker expression of T cells in peanut-sensitized donors after *in vitro* culture. A) a representative plot showing staining with tetramer and HLA-mismatch control. B) Quantification of tetramer-positive cells in peanut-sensitized, symptomatic (Sym) (n = 2) or non-symptomatic (Non-Sym) (n = 2) donors. In the non-symptomatic cohort, donors were tested with multiple tetramers, therefore a total of 4 data points are shown in this cohort. Median with interquartile range is shown. C) Integrin β7 expression and D) CRTh2 expression in tetramer positive cells from peanut-sensitized, symptomatic and non-symptomatic patients. Left panels show representative FACS plots. Right panels show graphs quantifying Integrin β7 and CRTh2 expression in tetramer+ cells from all samples tested. No statistical analysis was performed due to low sample size.

In the next series of experiments, we were interested in investigating whether differences could be detected in the phenotypes of peanut-specific T cells from symptomatic and non-symptomatic donors using tetramer reagents. The use of tetramers allows detection of peanut epitope-specific T cells on a single cell level. Using MHC class II binding assays, we determined peptide binding to MHC class II molecules that were expressed in our donor cohorts and for which tetramer reagents could be manufactured. A summary of tetramer reagents used is shown in **Table 3.** Based on donor-specific HLA expression, cell sample availability and tetramer allele reagent availability, we performed tetramer staining in 2 non-symptomatic and 2 symptomatic donors (**Table 3**). A representative tetramer staining with tetramer #2 and tetramer #1 as an HLA-mismatch control of donor 2377 is shown in **Fig 3A**. Quantification of tetramer-positive cells after *in vitro* expansion with single peptides revealed a trend for higher frequency of tetramer positive cells in symptomatic compared to non-symptomatic donors (**Fig 3B).**

**Table 3:** A summary of tetrameric reagents and respective donors tested.

Peptide #	Sequence	Allergen	Allele	MHC Binding (IC_50_ nM)	Tetramer	Donor tested
9	RRPFYSNAPLEIYVQ	Ara h 3	DRB1*09:01	24	#1	1198, 2384
10	QARQLKNNNPFKFFV	Ara h 3	DRB5*01:01	179	#2	2201, 2377
16	EFLAQAFQVDDRQIV	Ara h 3	DRB1*03:01	147	#3	2201
17	ARQQWELQGDRRCQS	Ara h 2	DRB1*03:01	1470	#4	2201

Next, we hypothesized that peanut-specific T cell responses in symptomatic donors are associated with a pathological type 2 phenotype, as previously reported [10, 19], whereas a more tolerogenic phenotype may be observed in non-symptomatics. To investigate this hypothesis, we performed co-staining of tetramer and surface markers that are associated with either a strong Th2 phenotype (CTRh2) or gut-homing (integrin β7), which may be associated with a more tolerogenic response as it has been shown that expression of integrin β7 plays a role in the regulation of gut-residential regulatory T cells [20] and immunoregulation of innate responses [21]. Analysis of peanut tetramer-positive T cells for their expression of the phenotype markers Integrin β7 (gut-homing marker) and CRTh2 (highly expressed on Th2 cells), revealed mild trends that indicate different T cell phenotypes for peanut-allergic and clinically symptomatic vs non-symptomatic patients. Expression of the gut-homing factor Integrin β7 was 2-fold lower in tetramer positive T cells from symptomatic patients compared to non-symptomatic patients (median 26.5% and 50%, respectively) (**Fig 3C**). In contrast, expression of CRTh2, a molecule associated with pathological type 2 T cell responses in allergy [19], exhibited the opposite pattern, with relatively high expression in symptomatic patients (median 6.5%) compared to non-symptomatics (median 0.4%) (**Fig 3D**). To assess if there were any differences in the level of expression, median fluorescent intensity was also assessed. No differences were observed (**S4 Fig).** While the data are preliminary and the sample size is very limited, it may suggest that differences exist in the phenotypes of peanut-specific T cells in symptomatic and non-symptomatic donors, which may be associated with observed differences in clinical reactivity between these two cohorts.

## Discussion

Peanut allergy is a common food allergy and can be associated with serious, sometimes even fatal adverse reactions. Here, we performed an analysis of the molecular targets and phenotype of peanut-specific T cell response to the allergens Ara h 1, Ara h 2 and Ara h 3. T cell epitope mapping in peanut allergic and non-allergic patients with HLA-matched peptide pools identified 74 T cell-reactive regions, 27 from Ara h 1, 4 from Ara h 2 and 43 from Ara h 3. Epitopic regions identified from Ara h 1 and 2 have been reported before [10–15]. However, to the best of our knowledge, this is the first report of Ara h 3-derived T cell-reactive epitopes in peanut-sensitized and non-sensitized patients. A major caveat of the HLA-matched epitope mapping approach performed is that it biases towards peptide binding of the HLA types expressed in the selected cohort. Consequently, it is important to highlight that the low number of epitopes identified in Ara h 2 is most likely due to the chosen approach rather than a reflection of reduced allergenicity compared to Ara h 1 and 3.

Overall, T cell responses from peanut allergics showed a higher IL-5:IFNγ ratio compared to non-allergics, consistent with studies in other allergy systems [22, 23]. Nevertheless, non-allergic donors also exhibited strong peanut-specific IL-5 production, which is surprising and may be due to the *in vitro* culture. The reduced IL-5:IFNγ ratio observed in non-allergics was mostly due to high levels of IFNγ rather than decreased IL-5, resulting in an overall more balanced Th1/Th2 response. It should further be noted that the two cohorts differ drastically in peanut exposure, which could further contribute to differences in peanut-specific T cell reactivity and phenotype.

Peanut sensitization, as defined by peanut-specific IgE titers >0.3 kU/L or positive skin prick test reactivity (diameter ≥ 3mm), is sometimes detected in patients who do not exhibit any clinical symptoms upon peanut ingestion. To learn more about the immunological reactivity on the T cell level in peanut-sensitized, symptomatic and non-symptomatic patients, and compared the peanut-specific T cell response in these two patient groups, with specific focus on response magnitude and polarization, epitope specificity and the T cell phenotype. While the use of peanut extract failed to detect any differences in peanut-specific T cell reactivity between the two cohorts, a significantly higher response in symptomatic patients (compared with non-symptomatic patients) was detected in response to a defined epitope pool composed of 19 peptides. In addition, a strong correlation between sIgE titers and P19 pool-specific T cell responses was observed, which was much less pronounced when compared to T cell reactivity against whole extract. Interestingly, we did not observe a difference in cytokine polarization between the two cohorts, indicating that the difference may be related to cell quantity rather than the functional response. Of note, the symptomatic cohort has a lower median age (23 years) compared to the non-symptomatic cohort (33 years), which may also be a factor contributing to the differences observed.

A previous study, which compared peanut allergic and non-allergic (non-sensitized) patients [10], reported a difference in magnitude between the two cohorts. Our data suggests that when defined epitopes are used to measure peanut-specific responses, non-symptomatic patients show a lower frequency of cytokine-producing, peanut-specific T cells compared to symptomatic patients, similar to what has been reported for non-sensitized donors. Interestingly, this difference in frequency between symptomatic and non-symptomatic donors was mostly accounted for by IL-10 and IL-17 production, and only a modest difference for IL-5 was observed. A recent study on single cell profiling of peanut-specific T cells reported the detection of multi-functional Th2 responses rather than a deficit in regulatory T cells among peanut-specific T cells in symptomatic donors[24]. In this context, the data may suggest that clinical reactivity is dictated by a potent, multi-functional T cell response, which may include a role for IL-17 as well as type 2 cytokines, rather than a dysfunctional regulatory response. Further studies are required to further elucidate the role of IL-17 and regulatory T cells in peanut allergy. Of note, this analysis was limited to IL-5, IL-10, IL-17 and IFNγ as representative cytokines for the major T cell subsets Th2, Tr1/Treg, Th17 and Th1. Further studies will have to be performed to determine if the difference in magnitude of the four cytokines extends to other cytokines or if a different read-out such as T cell activation or proliferation will return similar results.

Interrogation of the phenotype of tetramer positive T cells revealed that peanut-specific T cells from symptomatic patients tended to have higher CRTh2 expression compared to non-symptomatic donors. CRTh2 is associated with a Th2 phenotype implicated in allergic disease [10, 19]. In contrast, tetramer positive cells in non-symptomatic donors showed a trend for increased level of Integrin β7, a gut-homing factor that plays a role in the regulation of gut-residential regulatory T cells [20] and immune-regulation of innate responses [21]. Due to limited sample availability, tetramer experiments were performed in only four donors and therefore, no statistical analysis could be performed. Furthermore, the analysis performed does not compare T cells stained with the exact same tetramer, therefore the exact epitope specificity is different. Nevertheless, the identified trends are consistent with other studies that reported increased CRTh2 and decreased Integrin β7 expression in allergen-tetramer stained cells from allergic patients [10, 25]. Future studies in more expansive cohorts are required to confirm these trends.

Our findings show several notable differences in the peanut-specific T cell response from symptomatic and non-symptomatic donors, irrespective of their peanut sensitization. These data highlight that allergen component-specific reactivity is not limited to IgE and a better understanding of the T cell response may provide additional insights to understanding the different clinical manifestations observed in peanut allergy.

## Supporting information

S1 Fig Magnitude (SFC- spot forming cells) of IL-5 and IFNγ production in response to Ara h 1, 2 and 3-derived peptides that are recognized in both allergic (closed cirles) and non-allergic (open circles) individuals. Each dot represents a single peptide that elicited T cell reactivity in one or more donors. Statistical comparison across cohorts by Mann-Whitney test, within cohort by Wilcoxon signed rank test, two-tailed. ***- p<0.001,****<0.0001.
(TIF)

S2 Fig Magnitude of cytokine production (IL-5, IL-10, IL-17 and IFNγ) in response to A-D) P19 pool and E-H) peanut extract is shown as SFC (spot forming cells) per 10^6^ PBMC. Peanut sensitization status is indicated by IgE+. Sym = symptomatic (n = 15), Non-Sym = non-symptomatic (n = 14). Statistical comparison by Mann-Whitney test, one-tailed. *- p<0.05.
(TIF)

S3 Fig Correlation of antigen-specific T cell response and IgE titers. Correlation graphs showing T cell cytokine production against IgE titers. Statistical analysis is shown in Table 2. N = 29
(TIF)

S4 Fig Median Fluorescent intensity (MFI) of CRTh2 (left panel) and Integrin β7 (right panel) expression in tetramer positive cells. Graphs quantifying MFI of Integrin β7 and CRTh2 expression in tetramer+ cells from peanut-sensitized, symptomatic and non-symptomatic patients. No statistical analysis was performed due to low sample size.
(TIF)

S1 Table A summary of peanut allergen-derived T cell reactive peptides, number of donors tested and responding, and magnitude of T cell response (IL-5 and IFNγ producing cells). (XLSX)

## Abbreviations

IL: interleukin
PA: peanut allergy
HLA: human leukocyte antigen
Ig: immunoglobulin
IEDB: Immune Epitope DataBase
SFC: spot forming cell
PBMC: peripheral blood mononuclear cell
MHC: major histocompatibility complex
