# Supplementary figures and images for: Peanut-specific T cell responses in patients with different clinical reactivity
Source: PLoS One. 2018 Oct 10;13(10):e0204620. doi: 10.1371/journal.pone.0204620 (PMC6179248; doi:10.1371/journal.pone.0204620)

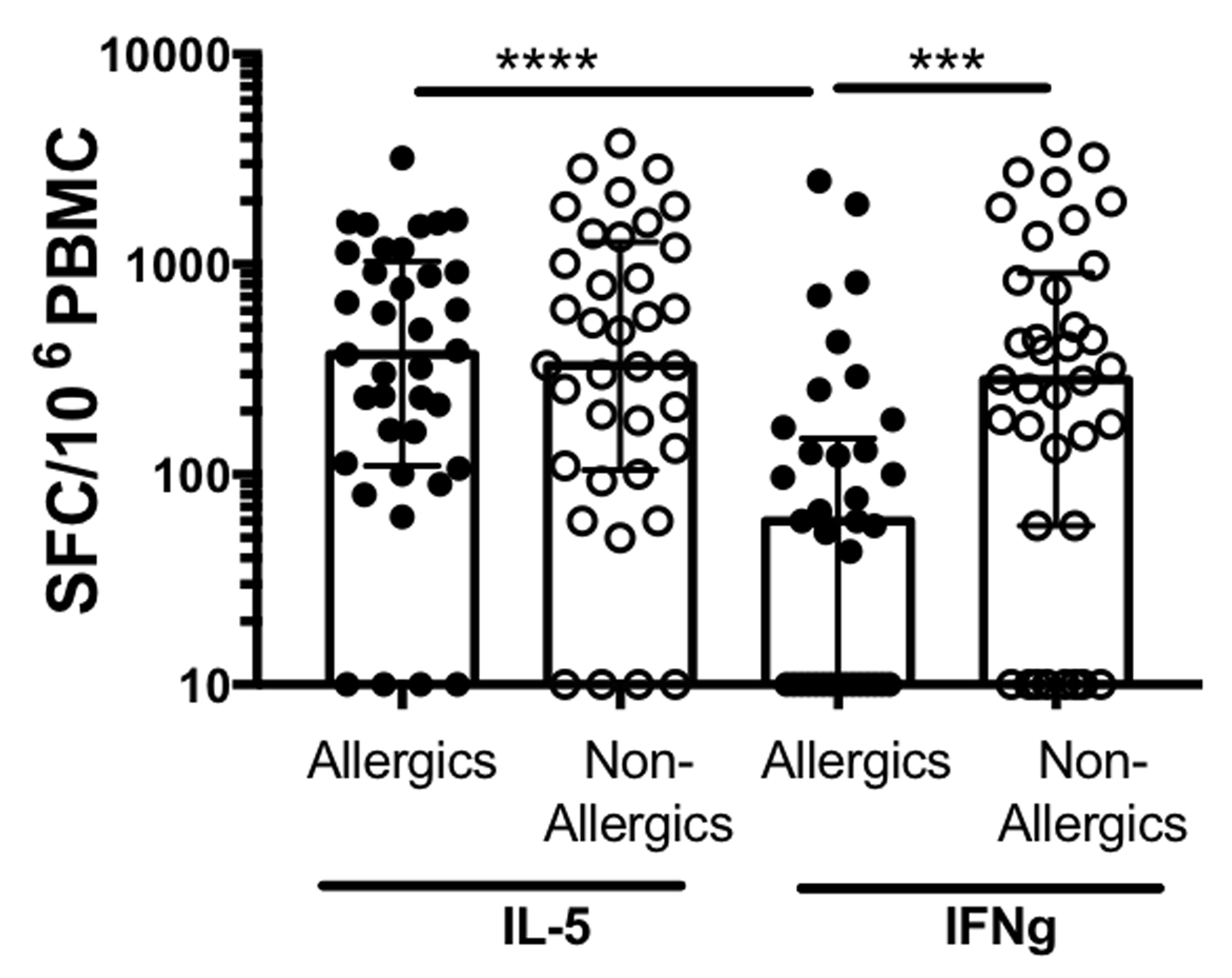

Supplement: S1 Fig — Each dot represents a single peptide that elicited T cell reactivity in one or more donors. Statistical comparison across cohorts by Mann-Whitney test, within cohort by Wilcoxon signed rank test, two-tailed. ***- p<0.001,****<0.0001. (TIF) [file pone.0204620.s001.tif]

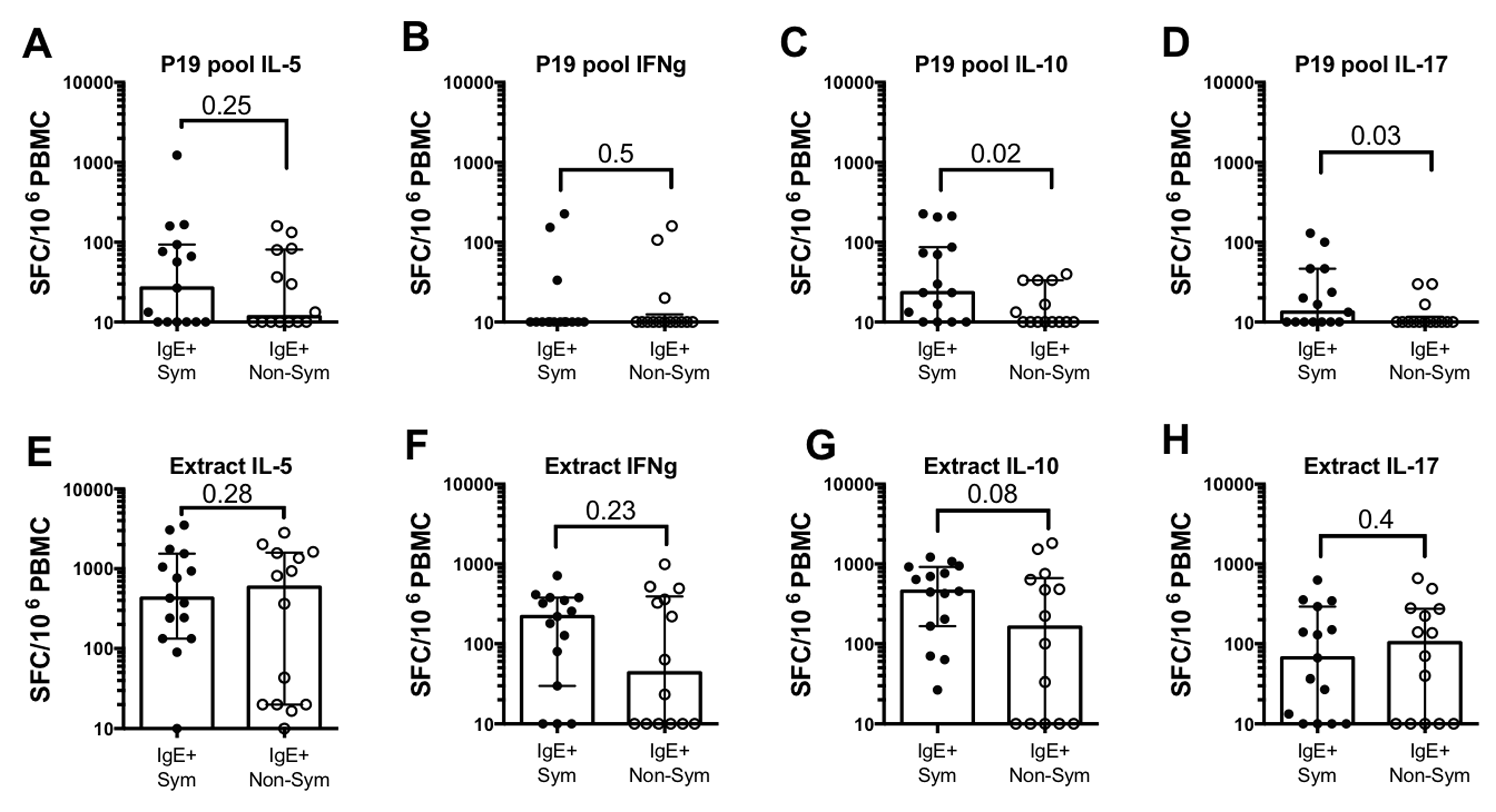

Supplement: S2 Fig — Peanut sensitization status is indicated by IgE+. Sym = symptomatic (n = 15), Non-Sym = non-symptomatic (n = 14). Statistical comparison by Mann-Whitney test, one-tailed. *- p<0.05. (TIF) [file pone.0204620.s002.tif]

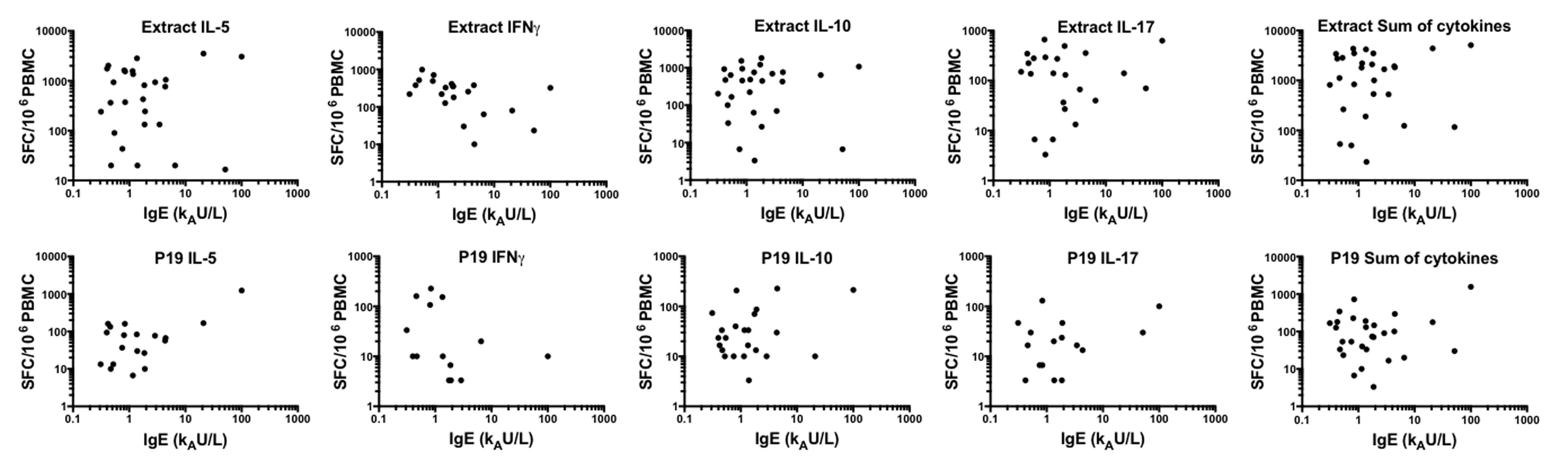

Supplement: S3 Fig — Correlation graphs showing T cell cytokine production against IgE titers. Statistical analysis is shown in Table 2. N = 29 (TIF) [file pone.0204620.s003.tif]

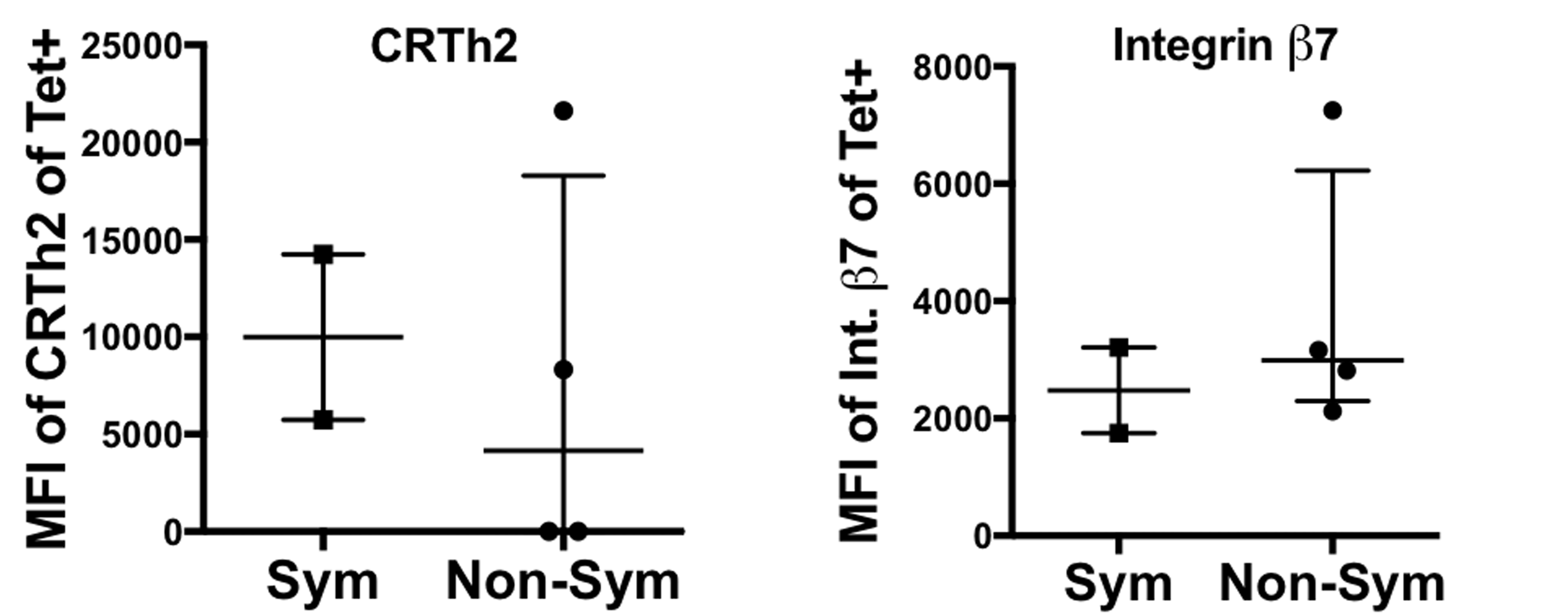

Supplement: S4 Fig — Median Fluorescent intensity (MFI) of CRTh2 (left panel) and Integrin β7 (right panel) expression in tetramer positive cells. Graphs quantifying MFI of Integrin β7 and CRTh2 expression in tetramer+ cells from peanut-sensitized, symptomatic and non-symptomatic patients. No statistical analysis was performed due to low sample size. (TIF) [file pone.0204620.s004.tif]
